# Measurement of *J*/*ψ* polarization in *pp* collisions at $\sqrt{s}=7\ \mathrm{TeV}$

**DOI:** 10.1140/epjc/s10052-013-2631-3

**Published:** 2013-11-09

**Authors:** The LHCb Collaboration, R. Aaij, C. Abellan Beteta, B. Adeva, M. Adinolfi, C. Adrover, A. Affolder, Z. Ajaltouni, J. Albrecht, F. Alessio, M. Alexander, S. Ali, G. Alkhazov, P. Alvarez Cartelle, A. A. Alves, S. Amato, S. Amerio, Y. Amhis, L. Anderlini, J. Anderson, R. Andreassen, R. B. Appleby, O. Aquines Gutierrez, F. Archilli, A. Artamonov, M. Artuso, E. Aslanides, G. Auriemma, S. Bachmann, J. J. Back, C. Baesso, V. Balagura, W. Baldini, R. J. Barlow, C. Barschel, S. Barsuk, W. Barter, Th. Bauer, A. Bay, J. Beddow, F. Bedeschi, I. Bediaga, S. Belogurov, K. Belous, I. Belyaev, E. Ben-Haim, M. Benayoun, G. Bencivenni, S. Benson, J. Benton, A. Berezhnoy, R. Bernet, M.-O. Bettler, M. van Beuzekom, A. Bien, S. Bifani, T. Bird, A. Bizzeti, P. M. Bjørnstad, T. Blake, F. Blanc, J. Blouw, S. Blusk, V. Bocci, A. Bondar, N. Bondar, W. Bonivento, S. Borghi, A. Borgia, T. J. V. Bowcock, E. Bowen, C. Bozzi, T. Brambach, J. van den Brand, J. Bressieux, D. Brett, M. Britsch, T. Britton, N. H. Brook, H. Brown, I. Burducea, A. Bursche, G. Busetto, J. Buytaert, S. Cadeddu, O. Callot, M. Calvi, M. Calvo Gomez, A. Camboni, P. Campana, D. Campora Perez, A. Carbone, G. Carboni, R. Cardinale, A. Cardini, H. Carranza-Mejia, L. Carson, K. Carvalho Akiba, G. Casse, L. Castillo Garcia, M. Cattaneo, Ch. Cauet, M. Charles, Ph. Charpentier, P. Chen, N. Chiapolini, M. Chrzaszcz, K. Ciba, X. Cid Vidal, G. Ciezarek, P. E. L. Clarke, M. Clemencic, H. V. Cliff, J. Closier, C. Coca, V. Coco, J. Cogan, E. Cogneras, P. Collins, A. Comerma-Montells, A. Contu, A. Cook, M. Coombes, S. Coquereau, G. Corti, B. Couturier, G. A. Cowan, D. C. Craik, S. Cunliffe, R. Currie, C. D’Ambrosio, P. David, P. N. Y. David, A. Davis, I. De Bonis, K. De Bruyn, S. De Capua, M. De Cian, J. M. De Miranda, L. De Paula, W. De Silva, P. De Simone, D. Decamp, M. Deckenhoff, L. Del Buono, D. Derkach, O. Deschamps, F. Dettori, A. Di Canto, H. Dijkstra, M. Dogaru, S. Donleavy, F. Dordei, A. Dosil Suárez, D. Dossett, A. Dovbnya, F. Dupertuis, R. Dzhelyadin, A. Dziurda, A. Dzyuba, S. Easo, U. Egede, V. Egorychev, S. Eidelman, D. van Eijk, S. Eisenhardt, U. Eitschberger, R. Ekelhof, L. Eklund, I. El Rifai, Ch. Elsasser, D. Elsby, A. Falabella, C. Färber, G. Fardell, C. Farinelli, S. Farry, V. Fave, D. Ferguson, V. Fernandez Albor, F. Ferreira Rodrigues, M. Ferro-Luzzi, S. Filippov, M. Fiore, C. Fitzpatrick, M. Fontana, F. Fontanelli, R. Forty, O. Francisco, M. Frank, C. Frei, M. Frosini, S. Furcas, E. Furfaro, A. Gallas Torreira, D. Galli, M. Gandelman, P. Gandini, Y. Gao, J. Garofoli, P. Garosi, J. Garra Tico, L. Garrido, C. Gaspar, R. Gauld, E. Gersabeck, M. Gersabeck, T. Gershon, Ph. Ghez, V. Gibson, V. V. Gligorov, C. Göbel, D. Golubkov, A. Golutvin, A. Gomes, H. Gordon, C. Gotti, M. Grabalosa Gándara, R. Graciani Diaz, L. A. Granado Cardoso, E. Graugés, G. Graziani, A. Grecu, E. Greening, S. Gregson, O. Grünberg, B. Gui, E. Gushchin, Yu. Guz, T. Gys, C. Hadjivasiliou, G. Haefeli, C. Haen, S. C. Haines, S. Hall, T. Hampson, S. Hansmann-Menzemer, N. Harnew, S. T. Harnew, J. Harrison, T. Hartmann, J. He, V. Heijne, K. Hennessy, P. Henrard, J. A. Hernando Morata, E. van Herwijnen, A. Hicheur, E. Hicks, D. Hill, M. Hoballah, C. Hombach, P. Hopchev, W. Hulsbergen, P. Hunt, T. Huse, N. Hussain, D. Hutchcroft, D. Hynds, V. Iakovenko, M. Idzik, P. Ilten, R. Jacobsson, A. Jaeger, E. Jans, P. Jaton, F. Jing, M. John, D. Johnson, C. R. Jones, C. Joram, B. Jost, M. Kaballo, S. Kandybei, M. Karacson, T. M. Karbach, I. R. Kenyon, U. Kerzel, T. Ketel, A. Keune, B. Khanji, O. Kochebina, I. Komarov, R. F. Koopman, P. Koppenburg, M. Korolev, A. Kozlinskiy, L. Kravchuk, K. Kreplin, M. Kreps, G. Krocker, P. Krokovny, F. Kruse, M. Kucharczyk, V. Kudryavtsev, T. Kvaratskheliya, V. N. La Thi, D. Lacarrere, G. Lafferty, A. Lai, D. Lambert, R. W. Lambert, E. Lanciotti, G. Lanfranchi, C. Langenbruch, T. Latham, C. Lazzeroni, R. Le Gac, J. van Leerdam, J.-P. Lees, R. Lefèvre, A. Leflat, J. Lefrançois, S. Leo, O. Leroy, T. Lesiak, B. Leverington, Y. Li, L. Li Gioi, M. Liles, R. Lindner, C. Linn, B. Liu, G. Liu, S. Lohn, I. Longstaff, J. H. Lopes, E. Lopez Asamar, N. Lopez-March, H. Lu, D. Lucchesi, J. Luisier, H. Luo, F. Machefert, I. V. Machikhiliyan, F. Maciuc, O. Maev, S. Malde, G. Manca, G. Mancinelli, U. Marconi, R. Märki, J. Marks, G. Martellotti, A. Martens, A. Martín Sánchez, M. Martinelli, D. Martinez Santos, D. Martins Tostes, A. Martynov, A. Massafferri, R. Matev, Z. Mathe, C. Matteuzzi, E. Maurice, A. Mazurov, J. McCarthy, A. McNab, R. McNulty, B. Meadows, F. Meier, M. Meissner, M. Merk, D. A. Milanes, M.-N. Minard, J. Molina Rodriguez, S. Monteil, D. Moran, P. Morawski, M. J. Morello, R. Mountain, I. Mous, F. Muheim, K. Müller, R. Muresan, B. Muryn, B. Muster, P. Naik, T. Nakada, R. Nandakumar, I. Nasteva, M. Needham, N. Neufeld, A. D. Nguyen, T. D. Nguyen, C. Nguyen-Mau, M. Nicol, V. Niess, R. Niet, N. Nikitin, T. Nikodem, A. Nomerotski, A. Novoselov, A. Oblakowska-Mucha, V. Obraztsov, S. Oggero, S. Ogilvy, O. Okhrimenko, R. Oldeman, M. Orlandea, J. M. Otalora Goicochea, P. Owen, A. Oyanguren, B. K. Pal, A. Palano, M. Palutan, J. Panman, A. Papanestis, M. Pappagallo, C. Parkes, C. J. Parkinson, G. Passaleva, G. D. Patel, M. Patel, G. N. Patrick, C. Patrignani, C. Pavel-Nicorescu, A. Pazos Alvarez, A. Pellegrino, G. Penso, M. Pepe Altarelli, S. Perazzini, D. L. Perego, E. Perez Trigo, A. Pérez-Calero Yzquierdo, P. Perret, M. Perrin-Terrin, K. Petridis, A. Petrolini, A. Phan, E. Picatoste Olloqui, B. Pietrzyk, T. Pilař, D. Pinci, S. Playfer, M. Plo Casasus, F. Polci, G. Polok, A. Poluektov, E. Polycarpo, D. Popov, B. Popovici, C. Potterat, A. Powell, J. Prisciandaro, A. Pritchard, C. Prouve, V. Pugatch, A. Puig Navarro, G. Punzi, W. Qian, J. H. Rademacker, B. Rakotomiaramanana, M. S. Rangel, I. Raniuk, N. Rauschmayr, G. Raven, S. Redford, M. M. Reid, A. C. dos Reis, S. Ricciardi, A. Richards, K. Rinnert, V. Rives Molina, D. A. Roa Romero, P. Robbe, E. Rodrigues, P. Rodriguez Perez, S. Roiser, V. Romanovsky, A. Romero Vidal, J. Rouvinet, T. Ruf, F. Ruffini, H. Ruiz, P. Ruiz Valls, G. Sabatino, J. J. Saborido Silva, N. Sagidova, P. Sail, B. Saitta, C. Salzmann, B. Sanmartin Sedes, M. Sannino, R. Santacesaria, C. Santamarina Rios, E. Santovetti, M. Sapunov, A. Sarti, C. Satriano, A. Satta, M. Savrie, D. Savrina, P. Schaack, M. Schiller, H. Schindler, M. Schlupp, M. Schmelling, B. Schmidt, O. Schneider, A. Schopper, M.-H. Schune, R. Schwemmer, B. Sciascia, A. Sciubba, M. Seco, A. Semennikov, K. Senderowska, I. Sepp, N. Serra, J. Serrano, P. Seyfert, M. Shapkin, I. Shapoval, P. Shatalov, Y. Shcheglov, T. Shears, L. Shekhtman, O. Shevchenko, V. Shevchenko, A. Shires, R. Silva Coutinho, T. Skwarnicki, N. A. Smith, E. Smith, M. Smith, M. D. Sokoloff, F. J. P. Soler, F. Soomro, D. Souza, B. Souza De Paula, B. Spaan, A. Sparkes, P. Spradlin, F. Stagni, S. Stahl, O. Steinkamp, S. Stoica, S. Stone, B. Storaci, M. Straticiuc, U. Straumann, V. K. Subbiah, S. Swientek, V. Syropoulos, M. Szczekowski, P. Szczypka, T. Szumlak, S. T’Jampens, M. Teklishyn, E. Teodorescu, F. Teubert, C. Thomas, E. Thomas, J. van Tilburg, V. Tisserand, M. Tobin, S. Tolk, D. Tonelli, S. Topp-Joergensen, N. Torr, E. Tournefier, S. Tourneur, M. T. Tran, M. Tresch, A. Tsaregorodtsev, P. Tsopelas, N. Tuning, M. Ubeda Garcia, A. Ukleja, D. Urner, U. Uwer, V. Vagnoni, G. Valenti, R. Vazquez Gomez, P. Vazquez Regueiro, S. Vecchi, J. J. Velthuis, M. Veltri, G. Veneziano, M. Vesterinen, B. Viaud, D. Vieira, X. Vilasis-Cardona, A. Vollhardt, D. Volyanskyy, D. Voong, A. Vorobyev, V. Vorobyev, C. Voß, H. Voss, R. Waldi, R. Wallace, S. Wandernoth, J. Wang, D. R. Ward, N. K. Watson, A. D. Webber, D. Websdale, M. Whitehead, J. Wicht, J. Wiechczynski, D. Wiedner, L. Wiggers, G. Wilkinson, M. P. Williams, M. Williams, F. F. Wilson, J. Wishahi, M. Witek, S. A. Wotton, S. Wright, S. Wu, K. Wyllie, Y. Xie, Z. Xing, Z. Yang, R. Young, X. Yuan, O. Yushchenko, M. Zangoli, M. Zavertyaev, F. Zhang, L. Zhang, W. C. Zhang, Y. Zhang, A. Zhelezov, A. Zhokhov, L. Zhong, A. Zvyagin

**Affiliations:** 1 CERN, 1211 Geneva 23, Switzerland; 2 Centro Brasileiro de Pesquisas Físicas (CBPF), Rio de Janeiro, Brazil; 3 Universidade Federal do Rio de Janeiro (UFRJ), Rio de Janeiro, Brazil; 4 Center for High Energy Physics, Tsinghua University, Beijing, China; 5 LAPP, Université de Savoie, CNRS/IN2P3, Annecy-Le-Vieux, France; 6 CNRS/IN2P3, LPC, Clermont Université, Université Blaise Pascal, Clermont-Ferrand, France; 7 CNRS/IN2P3, CPPM, Aix-Marseille Université, Marseille, France; 8 LAL, Université Paris-Sud, CNRS/IN2P3, Orsay, France; 9 LPNHE, Université Pierre et Marie Curie, Université Paris Diderot, CNRS/IN2P3, Paris, France; 10 Fakultät Physik, Technische Universität Dortmund, Dortmund, Germany; 11 Max-Planck-Institut für Kernphysik (MPIK), Heidelberg, Germany; 12 Physikalisches Institut, Ruprecht-Karls-Universität Heidelberg, Heidelberg, Germany; 13 School of Physics, University College Dublin, Dublin, Ireland; 14 Sezione INFN di Bari, Bari, Italy; 15 Sezione INFN di Bologna, Bologna, Italy; 16 Sezione INFN di Cagliari, Cagliari, Italy; 17 Sezione INFN di Ferrara, Ferrara, Italy; 18 Sezione INFN di Firenze, Firenze, Italy; 19 Laboratori Nazionali dell’INFN di Frascati, Frascati, Italy; 20 Sezione INFN di Genova, Genova, Italy; 21 Sezione INFN di Milano Bicocca, Milano, Italy; 22 Sezione INFN di Padova, Padova, Italy; 23 Sezione INFN di Pisa, Pisa, Italy; 24 Sezione INFN di Roma Tor Vergata, Roma, Italy; 25 Sezione INFN di Roma La Sapienza, Roma, Italy; 26 Henryk Niewodniczanski Institute of Nuclear Physics Polish Academy of Sciences, Kraków, Poland; 27 Faculty of Physics and Applied Computer Science, AGH - University of Science and Technology, Kraków, Poland; 28 National Center for Nuclear Research (NCBJ), Warsaw, Poland; 29 Horia Hulubei National Institute of Physics and Nuclear Engineering, Bucharest-Magurele, Romania; 30 Petersburg Nuclear Physics Institute (PNPI), Gatchina, Russia; 31 Institute of Theoretical and Experimental Physics (ITEP), Moscow, Russia; 32 Institute of Nuclear Physics, Moscow State University (SINP MSU), Moscow, Russia; 33 Institute for Nuclear Research of the Russian Academy of Sciences (INR RAN), Moscow, Russia; 34 Budker Institute of Nuclear Physics (SB RAS) and Novosibirsk State University, Novosibirsk, Russia; 35 Institute for High Energy Physics (IHEP), Protvino, Russia; 36 Universitat de Barcelona, Barcelona, Spain; 37 Universidad de Santiago de Compostela, Santiago de Compostela, Spain; 38 European Organization for Nuclear Research (CERN), Geneva, Switzerland; 39 Ecole Polytechnique Fédérale de Lausanne (EPFL), Lausanne, Switzerland; 40 Physik-Institut, Universität Zürich, Zürich, Switzerland; 41 Nikhef National Institute for Subatomic Physics, Amsterdam, The Netherlands; 42 Nikhef National Institute for Subatomic Physics and VU University Amsterdam, Amsterdam, The Netherlands; 43 NSC Kharkiv Institute of Physics and Technology (NSC KIPT), Kharkiv, Ukraine; 44 Institute for Nuclear Research of the National Academy of Sciences (KINR), Kyiv, Ukraine; 45 University of Birmingham, Birmingham, United Kingdom; 46 H.H. Wills Physics Laboratory, University of Bristol, Bristol, United Kingdom; 47 Cavendish Laboratory, University of Cambridge, Cambridge, United Kingdom; 48 Department of Physics, University of Warwick, Coventry, United Kingdom; 49 STFC Rutherford Appleton Laboratory, Didcot, United Kingdom; 50 School of Physics and Astronomy, University of Edinburgh, Edinburgh, United Kingdom; 51 School of Physics and Astronomy, University of Glasgow, Glasgow, United Kingdom; 52 Oliver Lodge Laboratory, University of Liverpool, Liverpool, United Kingdom; 53 Imperial College London, London, United Kingdom; 54 School of Physics and Astronomy, University of Manchester, Manchester, United Kingdom; 55 Department of Physics, University of Oxford, Oxford, United Kingdom; 56 Massachusetts Institute of Technology, Cambridge, MA United States; 57 University of Cincinnati, Cincinnati, OH United States; 58 Syracuse University, Syracuse, NY United States; 59 Pontifícia Universidade Católica do Rio de Janeiro (PUC-Rio), Rio de Janeiro, Brazil; 60 Institut für Physik, Universität Rostock, Rostock, Germany

## Abstract

An angular analysis of the decay *J*/*ψ*→*μ* ^+^ *μ* ^−^ is performed to measure the polarization of prompt *J*/*ψ* mesons produced in *pp* collisions at $\sqrt{s}=7~\mathrm{TeV}$. The dataset corresponds to an integrated luminosity of 0.37 fb^−1^ collected with the LHCb detector. The measurement is presented as a function of transverse momentum, *p*_T_, and rapidity, *y*, of the *J*/*ψ* meson, in the kinematic region 2<*p*_T_<15 GeV/*c* and 2.0<*y*<4.5.

## Introduction

Studies of *J*/*ψ* production in hadronic collisions provide powerful tests of QCD. In *pp* collisions, quarkonium resonances can be produced directly, through feed-down from higher quarkonium states (such as the *ψ*(2*S*) or *χ*_*c*_ resonances [1]), or via the decay of *b* hadrons. The first two production mechanisms are generically referred to as prompt production. The mechanism for prompt production is not yet fully understood and none of the available models adequately predicts the observed dependence of the *J*/*ψ* production cross-section and polarization on its transverse momentum *p*_T_ [1]. This paper describes the measurement of the polarization of the prompt *J*/*ψ* component in *pp* collisions at $\sqrt{s} = 7~\mathrm{TeV}$, using the dimuon decay mode. The measured polarization is subsequently used to update the LHCb measurement of the cross-section given in Ref. [2]. This improves the precision of the cross-section measurement significantly as the polarization and overall reconstruction efficiency are highly correlated.

The three polarization states of a *J*/*ψ* vector meson are specified in terms of a chosen coordinate system in the rest frame of the meson. This coordinate system is called the polarization frame and is defined with respect to a particular polarization axis. Defining the polarization axis to be the *Z*-axis, the *Y*-axis is chosen to be orthogonal to the production plane (the plane containing the *J*/*ψ* momentum and the beam axis) and the *X*-axis is oriented to create a right-handed coordinate system.

Several polarization frame definitions can be found in the literature. In the helicity frame [3] the polarization axis coincides with the flight direction of the *J*/*ψ* in the centre-of-mass frame of the colliding hadrons. In the Collins–Soper frame [4] the polarization axis is the direction of the relative velocity of the colliding beams in the *J*/*ψ* rest frame.

The angular decay distribution, apart from a normalization factor, is described by

1 $$\begin{aligned} \frac{d^2N}{d\cos\theta\,d\phi} \propto&1+\lambda_\theta\cos^2 \theta + \lambda_{\theta\phi}\sin2\theta\cos\phi \\ &{}+ \lambda_\phi\sin^2\theta\cos2\phi, \end{aligned}$$

where *θ* is the polar angle between the direction of the positive lepton and the chosen polarization axis, and *ϕ* is the azimuthal angle, measured with respect to the production plane. In this formalism, the polarization is completely longitudinal if the set of polarization parameters (*λ*_*θ*_, *λ*_*θϕ*_, *λ*_*ϕ*_) takes the values (−1,0,0) and it is completely transverse if it takes the values (1,0,0). In the zero polarization scenario the parameters are (0,0,0). In the general case, the values of (*λ*_*θ*_, *λ*_*θϕ*_, *λ*_*ϕ*_) depend on the choice of the spin quantization frame and different values can be consistent with the same underlying polarization states. However, the combination of parameters

2 $$ \lambda_{\mathrm{inv}} = \frac{\lambda_\theta+3\lambda_\phi}{1-\lambda _\phi} $$

is invariant under the choice of polarization frame [5, 6]. The natural polarization axis for the measurement is that where the lepton azimuthal angle distribution is symmetric (*λ*_*ϕ*_=*λ*_*θϕ*_=0) and *λ*_*θ*_ is maximal [7].

Several theoretical models are used to describe quarkonium production, predicting the values and the kinematic dependence of the cross-section and polarization. The color-singlet model (CSM) at leading order [8, 9] underestimates the *J*/*ψ* production cross-section by two orders of magnitude [2, 10] and predicts significant transverse polarization. Subsequent calculations at next-to-leading order and at next-to-next-to-leading order change these predictions dramatically. The cross-section prediction comes close to the observed values and the polarization is expected to be large and longitudinal [11–14]. Calculations performed in the framework of non-relativistic quantum chromodynamics (NRQCD), where the $c\bar{c}$ pair can be produced in color-octet states (color-octet model, COM [15–17]), can explain the shape and magnitude of the measured cross-section as a function of *p*_T_. COM predicts a dependence of the *J*/*ψ* polarization on the *p*_T_ of the *J*/*ψ* meson. In the low *p*_T_ region (*p*_T_<*M*(*J*/*ψ*)/*c* with *M*(*J*/*ψ*) the mass of the *J*/*ψ* meson), where the gluon fusion process dominates, a small longitudinal polarization is expected [18]. For *p*_T_≫*M*(*J*/*ψ*), where gluon fragmentation dominates, the leading order predictions [19, 20] and next-to-leading order calculations [21] suggest a large transverse component of the *J*/*ψ* polarization.

The polarization for inclusive *J*/*ψ* production (including the feed-down from higher charmonium states) in hadronic interactions has been measured by several experiments at Fermilab [22], Brookhaven [23] and DESY [24]. The CDF experiment, in $p\bar{p}$ collisions at $\sqrt{s}=1.96~\mathrm{TeV}$, measured a small longitudinal *J*/*ψ* polarization, going to zero at small *p*_T_. This measurement is in disagreement with the COM calculations and does not support the conclusion that the color-octet terms dominate the *J*/*ψ* production in the high *p*_T_ region. The PHENIX experiment measured the *J*/*ψ* polarization in *pp* collisions at $\sqrt{s}=200~\mathrm{GeV}$, for *p*_T_<3 GeV/*c*. The HERA-B experiment studied *J*/*ψ* polarization in 920 GeV/*c* fixed target proton-nucleus (*p*-*C* and *p*-*W*) collisions. The explored kinematic region is defined for *p*_T_<5.4 GeV/*c* and Feynman variable *x*_F_ between −0.34 and 0.14. Also in these cases a small longitudinal polarization is observed. Recently, at the LHC, ALICE [25] and CMS [26] have measured the *J*/*ψ* polarization in *pp* collisions at $\sqrt{s} = 7~\mathrm{TeV}$, in the kinematic ranges of 2<*p*_T_<8 GeV/*c*, 2.5<*y*<4.0, and 14<*p*_T_<70 GeV/*c*, |*y*|<1.2, respectively. The ALICE collaboration finds a small longitudinal polarization vanishing at high values of *p*_T_,^1^ while the CMS results do not show evidence of large transverse or longitudinal polarizations.

The analysis presented here is performed by fitting the efficiency-corrected angular distribution of the data. Given the forward geometry of the LHCb experiment, the polarization results are presented in the helicity frame and, as a cross-check, in the Collins–Soper frame. The polarization is measured by performing a two-dimensional angular analysis considering the distribution given in Eq. (1) and using an unbinned maximum likelihood fit. To evaluate the detector acceptance, reconstruction and trigger efficiency, fully simulated events are used. The measurement is performed in six bins of *J*/*ψ* transverse momentum and five rapidity bins. The edges of the bins in *J*/*ψ* *p*_T_ and *y* are defined respectively as [2, 3, 4, 5, 7, 10, 15] GeV/*c* in *J*/*ψ* *p*_T_ and [2.0, 2.5, 3.0, 3.5, 4.0, 4.5] in *J*/*ψ* *y*.

The remainder of the paper is organized as following. In Sect. 2 a brief description of the LHCb detector and the data sample used for the analysis is given. In Sect. 3 the signal selection is defined. In Sects. 4 and 5 respectively, the fit procedure to the angular distribution and the contributions to the systematic uncertainties on the measurement are described. The results are presented in Sect. 6 and in Sect. 7 the update of the *J*/*ψ* cross-section, including the polarization effect, is described. Finally in Sect. 8 conclusions are drawn.

## LHCb detector and data sample

The LHCb detector [27] is a single-arm forward spectrometer covering the pseudorapidity range 2<*η*<5, designed for the study of hadrons containing *b* or *c* quarks. A right-handed Cartesian coordinate system is used, centred on the nominal *pp* collision point with *z* pointing downstream along the nominal beam axis and *y* pointing upwards. The detector includes a high precision tracking system consisting of a silicon-strip vertex detector surrounding the *pp* interaction region, a large-area silicon-strip detector located upstream of a dipole magnet with a bending power of about 4 Tm, and three stations of silicon-strip detectors and straw drift tubes placed downstream. The combined tracking system provides momentum measurement with relative uncertainty that varies from 0.4 % at 5 GeV/*c* to 0.6 % at 100 GeV/*c*, and impact parameter resolution of 20 μm for tracks with high *p*_T_. Charged hadrons are identified using two ring-imaging Cherenkov detectors. Photon, electron and hadron candidates are identified by a calorimeter system consisting of scintillating-pad and pre-shower detectors, an electromagnetic calorimeter and a hadronic calorimeter. Muons are identified by a system composed of alternating layers of iron and multiwire proportional chambers [28].

The trigger [29] consists of a hardware stage, based on information from the calorimeter and muon systems, followed by a software stage, which applies a full event reconstruction. Candidate events are selected by the hardware trigger requiring the *p*_T_ of one muon to be larger than 1.48 GeV/*c*, or the products of the *p*_T_ of the two muons to be larger than 1.68 (GeV/*c*)^2^. In the subsequent software trigger [29], two tracks with *p*_T_>0.5 GeV/*c* and momentum *p*>6 GeV/*c* are required to be identified as muons and the invariant mass of the two muon tracks is required to be within ±120 MeV/*c* ^2^ of the nominal mass of the *J*/*ψ* meson [30]. The data used for this analysis correspond to an integrated luminosity of 0.37 fb^−1^ of *pp* collisions at a center-of-mass energy of $\sqrt{s}= 7~\mathrm{TeV}$, collected by the LHCb experiment in the first half of 2011. The period of data taking has been chosen to have uniform trigger conditions.

In the simulation, *pp* collisions are generated using Pythia 6.4 [31] with a specific LHCb configuration [32]. Decays of hadronic particles are described by EvtGen [33], in which final state radiation is generated using Photos [34]. The interaction of the generated particles with the detector and its response are implemented using the Geant4 toolkit [35, 36] as described in Ref. [37]. The prompt charmonium production is simulated in Pythia according to the leading order color-singlet and color-octet mechanisms.

## Signal selection

The selection requires that at least one primary vertex is reconstructed in the event. Candidate *J*/*ψ* mesons are formed from pairs of opposite-sign tracks reconstructed in the tracking system. Each track is required to have *p*_T_>0.75 GeV/*c* and to be identified as a muon. The two muons must originate from a common vertex and the *χ* ^2^ probability of the vertex fit must be greater than 0.5 %.

**Fig. 1:**
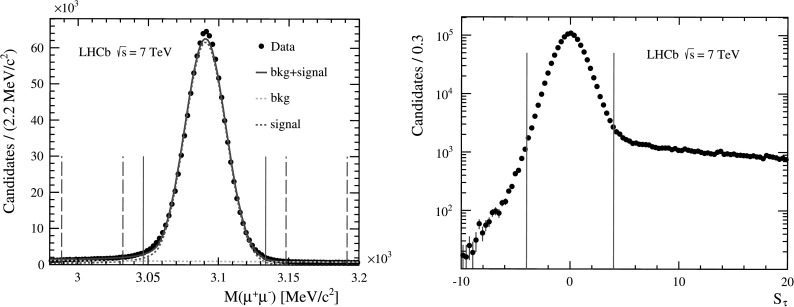
(*Left*) Invariant mass distribution of muon pairs passing the selection criteria. In the plot, *J*/*ψ* candidates are required to have 5<*p*_T_<7 GeV/*c* and 3.0<*y*<3.5. The *solid* (*dashed*) *vertical lines* indicate the signal (sideband) regions. (*Right*) Pseudo decay-time significance (*S*_*τ*_) distribution for background subtracted *J*/*ψ* candidates in the same kinematic bin. The *solid vertical lines* indicate the *S*_*τ*_ selection region. The right tail of the distribution is due to *J*/*ψ* production through the decay of *b* hadrons

In Fig. 1 (left), the invariant mass distribution of *J*/*ψ* candidates for 5<*p*_T_<7 GeV/*c* and 3.0<*y*<3.5 is shown as an example. A fit to the mass distribution has been performed using a Crystal Ball function [38] for the signal and a linear function for the background, whose origin is combinatorial. The Crystal Ball parameter describing the threshold of the radiative tail is fixed to the value obtained in the simulation. The Crystal Ball peak position and resolution determined in the fit shown in Fig. 1 (left) are respectively *μ*=3090.5 MeV/*c* ^2^ and *σ*=14.6 MeV/*c* ^2^. The signal region is defined as [*μ*−3*σ*,*μ*+3*σ*] and the two sideband regions as [*μ*−7*σ*,*μ*−4*σ*] and [*μ*+4*σ*,*μ*+7*σ*] in the mass distribution.

Prompt *J*/*ψ* mesons and *J*/*ψ* mesons from *b*-hadron decays can be discriminated by the pseudo-decay-time *τ*, which is defined as:

3 $$ \tau= \frac{(z_{J/\psi} - z_{\mathrm{PV}})M(J/\psi)}{p_{z}}, $$

where *z*_*J*/*ψ*_ and *z*_PV_ are the positions of the *J*/*ψ* decay vertex and the associated primary vertex along the *z*-axis, *M*(*J*/*ψ*) is the nominal *J*/*ψ* mass and *p*_*z*_ is the measured *z* component of the *J*/*ψ* momentum in the center-of-mass frame of the *pp* collision. For events with several primary vertices, the one which is closest to the *J*/*ψ* vertex is used. The uncertainty *σ*_*τ*_ is calculated for each candidate using the measured covariance matrix of *z*_*J*/*ψ*_ and *p*_*z*_ and the uncertainty of *z*_PV_. The bias induced by not refitting the primary vertex removing the two tracks from the reconstructed *J*/*ψ* meson is found to be negligible [2]. The pseudo decay-time significance *S*_*τ*_ is defined as *S*_*τ*_=*τ*/*σ*_*τ*_. In order to suppress the component of *J*/*ψ* mesons from *b*-hadron decays, it is required that |*S*_*τ*_|<4. With this requirement, the fraction of *J*/*ψ* from *b*-hadron decays reduces from about 15 % to about 3 %. The distribution of the pseudo-decay-time significance in one kinematic bin is shown in Fig. 1 (right).

## Polarization fit

**Fig. 2:**
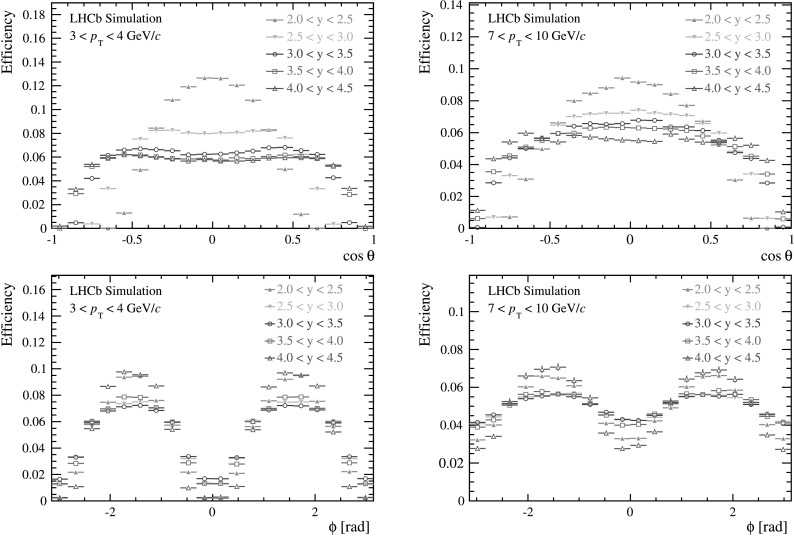
Global efficiency (area normalized to unity) as a function of (*top*) cos*θ* and (*bottom*) *ϕ* for (*left*) 3<*p*_T_<4 GeV/*c* and for (*right*) 7<*p*_T_<10 GeV/*c* of *J*/*ψ* mesons in the helicity frame. The efficiency is determined from simulation

The polarization parameters are determined from a fit to the angular distribution (cos*θ*,*ϕ*) of the *J*/*ψ*→*μ* ^+^ *μ* ^−^ decay. The knowledge of the efficiency as a function of the angular variables (cos*θ*,*ϕ*) is crucial for the analysis. The detection efficiency *ϵ* includes geometrical, detection and trigger efficiencies and is obtained from a sample of simulated unpolarized *J*/*ψ* mesons decaying in the *J*/*ψ*→*μ* ^+^ *μ* ^−^ channel, where the events are divided in bins of *p*_T_ and *y* of the *J*/*ψ* meson. The efficiency is studied as a function of four kinematic variables: *p*_T_ and *y* of the *J*/*ψ* meson, and cos*θ* and *ϕ* of the positive muon. As an example, Fig. 2 shows the efficiency as a function of cos*θ* (integrated over *ϕ*) and *ϕ* (integrated over cos*θ*) respectively, for two different bins of *p*_T_ and all five bins of *y*. The efficiency is lower for cos*θ*≈±1, as one of the two muons in this case has a small momentum in the center-of-mass frame of the *pp* collision and is often bent out of the detector acceptance by the dipole field of the magnet. The efficiency is also lower for |*ϕ*|≈0 or *π*, because one of the two muons often escapes the LHCb detector acceptance.

To fit the angular distribution in Eq. (1), a maximum likelihood (ML) approach is used. The logarithm of the likelihood function, for data in each *p*_T_ and *y* bin, is defined as

4 $$\begin{aligned} \log L =&\sum^{N_{\mathrm{tot}}}_{i=1}w_i \\ &{}\times \log \biggl[\frac{P(\cos\theta_{i},\phi_{i}\vert\lambda_{\theta}, \lambda_{\theta\phi}, \lambda_{\phi})\,\epsilon(\cos\theta_{i},\phi _{i})}{N(\lambda_{\theta}, \lambda_{\theta\phi}, \lambda_{\phi})} \biggr] \end{aligned}$$

5 $$\begin{aligned} =&\sum^{N_{\mathrm{tot}}}_{i=1}w_i\times \log \biggl[\frac{P(\cos\theta_{i},\phi_{i}\vert\lambda_{\theta}, \lambda_{\theta\phi}, \lambda_{\phi})}{N(\lambda_{\theta}, \lambda_{\theta\phi}, \lambda_{\phi})} \biggr] \\ &{}+ \sum^{N_{\mathrm {tot}}}_{i=1}w_{i} \times \log \bigl[\epsilon(\cos\theta_{i},\phi_{i}) \bigr] , \end{aligned}$$

where

$$\begin{aligned} &{P(\cos\theta_{i},\phi_{i}\vert\lambda_{\theta}, \lambda_{\theta\phi}, \lambda_{\phi})}\\ &{\quad = 1+\lambda_\theta\cos^2 \theta_{i} + \lambda_{\theta\phi}\sin2\theta_{i} \cos\phi_{i} + \lambda_\phi\sin^2 \theta_{i} \cos2\phi_{i},} \end{aligned}$$

*w*_*i*_ are weighting factors and the index *i* runs over the number of the candidates, *N*_tot_. The second sum in Eq. (5) can be ignored in the fit as it has no dependence on the polarization parameters. *N*(*λ*_*θ*_,*λ*_*θϕ*_,*λ*_*ϕ*_) is a normalization integral, defined as

6 $$\begin{aligned} &{N(\lambda_{\theta},\lambda_{\theta\phi}, \lambda_{\phi})} \\ &{\quad = \int d\varOmega\, P(\cos\theta,\phi\vert \lambda_{\theta}, \lambda_{\theta\phi}, \lambda_{\phi})\times \epsilon(\cos\theta,\phi).} \end{aligned}$$

In the simulation where *J*/*ψ* mesons are generated unpolarized, the (cos*θ*,*ϕ*) two-dimensional distribution of selected candidates is the same as the efficiency *ϵ*(cos*θ*,*ϕ*), so Eq. (6) can be evaluated by summing *P*(cos*θ*_*i*_,*ϕ*_*i*_|*λ*_*θ*_,*λ*_*θϕ*_,*λ*_*ϕ*_) over the *J*/*ψ* candidates in the simulated sample. The normalization *N*(*λ*_*θ*_,*λ*_*θϕ*_,*λ*_*ϕ*_) depends on all three polarization parameters. The weighting factor *w*_*i*_ is chosen to be +1 (−1) if a candidate falls in the signal region (sideband regions) shown in Fig. 1. In this way the background component in the signal window is subtracted on a statistical basis.^2^ For this procedure it is assumed that the angular distribution (cos*θ*,*ϕ*) of background events in the signal region is similar to that of the events in sideband regions, and that the mass distribution of the background is approximately linear.

The method used for the measurement of the polarization is tested by measuring the *J*/*ψ* polarization in two simulated samples with a fully transverse and fully longitudinal polarization, respectively. In both cases the results reproduce the simulation input within the statistical sensitivity.

To evaluate the normalization function *N*(*λ*_*θ*_,*λ*_*θϕ*_,*λ*_*ϕ*_) on the simulated sample of unpolarized *J*/*ψ* mesons, we rely on the correct simulation of the efficiency. In order to cross check the reliability of the efficiency obtained from the simulation, the control-channel *B* ^+^→*J*/*ψK* ^+^ is studied. The choice of this channel is motivated by the fact that, due to angular momentum conservation, the *J*/*ψ* must be longitudinally polarized and any difference between the angular distributions measured in data and in the simulation must be due to inaccuracies in the simulation.

**Fig. 3:**
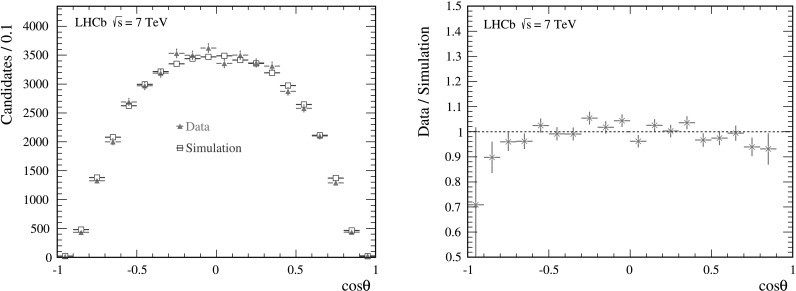
(*Left*) Distributions of cos*θ* in the helicity frame for *J*/*ψ* mesons from *B* ^+^→*J*/*ψK* ^+^ decays in data (*open circles*) and simulated sample (*open squares*) after the weighting based on the *B* ^+^ and *J*/*ψ* kinematics and (*right*) their ratio

To compare the kinematic variables of the muons in data and simulation, a first weighting procedure is applied to the simulated sample to reproduce the *B* ^+^ and *J*/*ψ* kinematics in the data. In Fig. 3, cos*θ* distributions for *B* ^+^→*J*/*ψK* ^+^ candidates for data and simulation are shown, as well as their ratio. A small difference between the distributions for data and simulation is observed, which is attributed to an overestimation of the efficiency in the simulation for candidates with values of |cos*θ*|≈1. To correct for the acceptance difference, an additional event weighting is applied where the weighting factors are obtained by comparing the two-dimensional muon *p*_T_ and *y* distribution in the center-of-mass frame of *pp* collisions in data and simulation. This weighting corrects for the observed disagreement in the cos*θ* distribution. The weights as a function of muon *p*_T_ and *y* obtained from the *B* ^+^→*J*/*ψK* ^+^ sample are subsequently applied in the same way to the simulated prompt *J*/*ψ* sample, which is used to determine the efficiency for the polarization measurement.

## Systematic uncertainties

The largest systematic uncertainty is related to the determination of the efficiency and to the weighting procedure used to correct the simulation, using the *B* ^+^→*J*/*ψK* ^+^ control channel. The weighting procedure is performed in bins of *p*_T_ and *y* of the two muons and, due to the limited number of candidates in the control channel, the statistical uncertainties of the correction factors are sizeable (from 1.3 % up to 25 %, depending on the bin). To propagate these uncertainties to the polarization results, the following procedure is used. For each muon (*p*_T_,*y*) bin, the weight is changed by one standard deviation, leaving all other weights at their nominal values. This new set of weights is used to redetermine the detector efficiency and then perform a new fit of the polarization parameters. The difference of the obtained parameters with respect to the nominal polarization result is considered as the contribution of this muon (*p*_T_,*y*) bin to the uncertainty. The total systematic uncertainty is obtained by summing all these independent contributions in quadrature. In the helicity frame, the average absolute uncertainty over all the *J*/*ψ* (*p*_T_,*y*) bins due to this effect is 0.067 on *λ*_*θ*_.

Concerning the background subtraction, the choice of the sidebands and the background model are checked. A systematic uncertainty is evaluated by comparing the nominal results for the polarization parameters, and those obtained using only the left or the right sideband, or changing the background fit function (as alternatives to the linear function, exponential and polynomial functions are used). In both cases the maximum variation with respect to the nominal result is assigned as systematic uncertainty. Typically, the absolute size of this effect is 0.012 on *λ*_*θ*_ for *p*_T_>5 GeV/*c*.

The effect of the (*p*_T_,*y*) binning for the *J*/*ψ* meson could also introduce an uncertainty, due to the difference of the *J*/*ψ* kinematic distributions between data and simulation within the bins. To investigate this effect, each bin is divided in four sub-bins (2×2) and the polarization parameters are calculated in each sub-bin. The weighted average of the results in the four sub-bins is compared with the nominal result and the difference is quoted as the systematic uncertainty. As expected, this effect is particularly important in the rapidity range near the LHCb acceptance boundaries, where the efficiency has a strong dependence on the kinematic properties of the *J*/*ψ* meson. It however depends on *p*_T_ only weakly and the average effect on *λ*_*θ*_ is 0.018 (absolute).

Two systematic uncertainties related to the cut on the *J*/*ψ* decay time significance are evaluated. The first is due to the residual *J*/*ψ* candidates from *b*-hadron decays, 3 % on average and up to 5 % in the highest *p*_T_ bins, that potentially have different polarization. The second is due to the efficiency difference in the *S*_*τ*_ requirement in data and simulation. The average size of these effects, over the *J*/*ψ* (*p*_T_,*y*), is 0.012.

The limited number of events in the simulation sample, used to evaluate the normalization integrals of Eq. (6), is a source of uncertainty. This effect is evaluated by simulating a large number of pseudo-experiments and the average absolute size is 0.015.

Finally, the procedure used to statistically subtract the background introduces a statistical uncertainty, not included in the standard likelihood maximization uncertainty. A detailed investigation shows that it represents a tiny correction to the nominal statistical uncertainty, reported in Tables 2 and 3.

The main contributions to the systematic uncertainties on *λ*_*θ*_ are summarized in Table 1 for the helicity and the Collins–Soper frames. While all uncertainties are evaluated for every *p*_T_ and *y* bin separately, we quote for the individual contributions only the average, minimum and maximum values. The systematic uncertainties on *λ*_*θϕ*_ and *λ*_*ϕ*_ are similar to each other and a factor two lower than those for *λ*_*θ*_. Apart from the binning and the simulation sample size effects, the uncertainties of adjacent kinematic bins are strongly correlated.

**Table 1:** Main contributions to the absolute systematic uncertainty on the parameter *λ*_*θ*_ in the helicity and Collins–Soper frames. While the systematic uncertainties are evaluated separately for all *p*_T_ and *y* bins, we give here only the average, the minimum and the maximum values of all bins

Source	Helicity frame average (min.–max.)	Collins–Soper frame average (min.–max.)
Acceptance	0.067 (0.045–0.173)	0.044 (0.025–0.185)
Binning effect	0.018 (0.001–0.165)	0.016 (0.001–0.129)
Simulation sample size	0.015 (0.005–0.127)	0.015 (0.007–0.170)
Sideband subtraction	0.016 (0.001–0.099)	0.029 (0.001–0.183)
*b*-hadron contamination	0.012 (0.002–0.019)	0.006 (0.002–0.029)

To quote the global systematic uncertainty (Tables 2 and 3) in each kinematic bin of the *J*/*ψ* meson, the different contributions for each bin are considered to be uncorrelated and are added in quadrature.

## Results

**Fig. 4:**
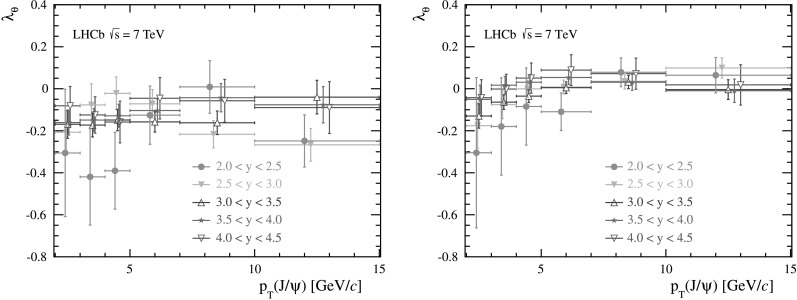
Measurements of *λ*_*θ*_ in bins of *p*_T_ for five rapidity bins in (*left*) the helicity frame and (*right*) the Collins–Soper frame. The *error bars* represent the statistical and systematic uncertainties added in quadrature. The data points are shifted slightly horizontally for different rapidities to improve visibility

The fit results for the three parameters *λ*_*θ*_, *λ*_*θϕ*_ and *λ*_*ϕ*_, with their uncertainties, are reported in Tables 2 and 3 for the helicity frame and the Collins–Soper frame, respectively. The parameter *λ*_*θ*_ is also shown in Fig. 4 as a function of the *p*_T_ of the *J*/*ψ* meson, for different *y* bins.

The polarization parameters *λ*_*ϕ*_ and *λ*_*θϕ*_ in the helicity frame are consistent with zero within the uncertainties. Following the discussion in Sect. 1, the helicity frame represents the natural frame for the polarization measurement in our experiment and the measured *λ*_*θ*_ parameter is an indicator of the *J*/*ψ* polarization, since it is equal to the invariant parameter defined in Eq. (2).

The measured value of *λ*_*θ*_ shows a small longitudinal polarization. A weighted average is calculated over all the (*p*_T_,*y*) bins, where the weights are chosen according to the number of events in each bin in the data sample. The average is *λ*_*θ*_=−0.145±0.027. The uncertainty is statistical and systematic uncertainties added in quadrature. Since the correlations of the systematic uncertainties are observed to be relevant only between adjacent kinematic bins, when quoting the average uncertainty, we assume the different kinematic bins are uncorrelated, apart from the adjacent ones, which we treat fully correlated.

A cross-check of the results is performed by repeating the measurement in the Collins–Soper reference frame (see Sect. 1). As LHCb is a forward detector, the Collins–Soper and helicity frames are kinematically quite similar, especially in the low *p*_T_ and *y* regions. Therefore, the polarization parameters obtained in Collins–Soper frame are expected to be similar to those obtained in the helicity frame, except at high *p*_T_ and low *y* bins. Calculating the frame-invariant variable, according to Eq. (2), the measurements performed in the two frames are in agreement within the uncertainty.

**Fig. 5:**
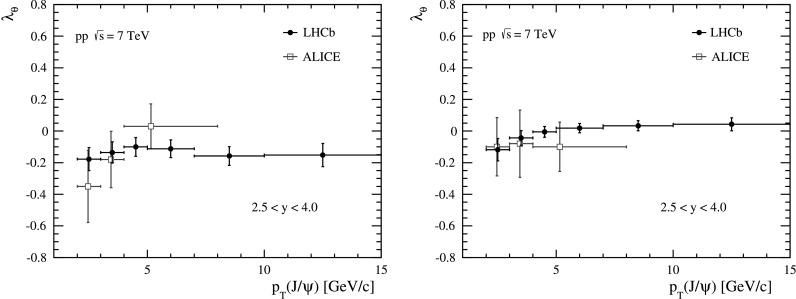
Comparison of LHCb and ALICE results for *λ*_*θ*_ in different *p*_T_ bins integrating over the rapidity range 2.5<*y*<4.0 in (*left*) the helicity frame and (*right*) the Collins–Soper frame. *Error bars* represent the statistical and systematic uncertainties added in quadrature

The results can be compared to those obtained by other experiments at different values of $\sqrt{s}$. Measurements by CDF [22], PHENIX [23] and HERA-B [24], also favor a negative value for *λ*_*θ*_. The HERA-B experiment has also published results on *λ*_*ϕ*_ and *λ*_*θϕ*_, which are consistent with zero. At the LHC, the ALICE [25] and the CMS [26] collaboration studied the *J*/*ψ* polarization in *pp* collisions at $\sqrt{s} = 7~\mathrm{TeV}$. The CMS results, determined in a different kinematic range, disfavor large transverse or longitudinal polarizations. The analysis by ALICE is based on the cos*θ* and *ϕ* projections and thus only determines *λ*_*θ*_ and *λ*_*ϕ*_. Furthermore it also includes *J*/*ψ* mesons from *b*-hadron decays. The measurement has been performed in bins of *J*/*ψ* transverse momentum integrating over the rapidity in a range very similar to that of LHCb, being 2<*p*_T_<8 GeV/*c* and 2.5<*y*<4.0. To compare our results with the ALICE measurements, averages over the *y* region are used for the different *p*_T_ bins and good agreement is found for *λ*_*θ*_ and *λ*_*ϕ*_. The comparison for *λ*_*θ*_ is shown in Fig. 5 for the helicity and Collins–Soper frames, respectively.

**Fig. 6:**
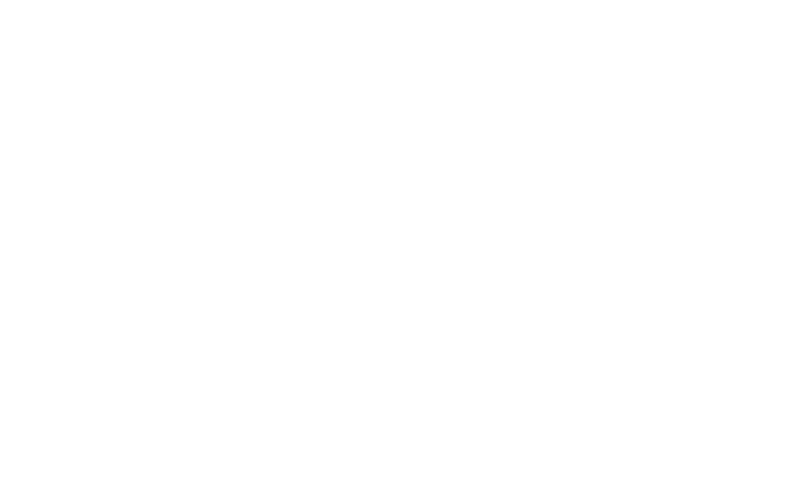
Comparison of LHCb prompt *J*/*ψ* polarization measurements of *λ*_*θ*_ with direct NLO color singlet (*magenta diagonal lines* [39]) and three different NLO NRQCD (*blue diagonal lines* (1) [39], *red vertical lines* (2) [40] and *green hatched* (3) [41, 42]) predictions as a function of the *p*_T_ of the *J*/*ψ* meson in the rapidity range 2.5<*y*<4.0 in the helicity frame (Color figure online)

In Fig. 6 our measurements of *λ*_*θ*_ are compared with the NLO CSM [39] and NRQCD predictions of Refs. [39, 40] and [41, 42]. The comparison is done in the helicity frame and as a function of the *p*_T_ of the *J*/*ψ* meson (integrating over 2.5<*y*<4.0). The theoretical calculations in Refs. [39, 40] and [41, 42] use different selections of experimental data to evaluate the non-perturbative matrix elements. Our results are not in agreement with the CSM predictions and the best agreement is found between the measured values and the NRQCD predictions of Refs. [41, 42]. It should be noted that our analysis includes a contribution from feed-down, while the theoretical computations from CSM and NRQCD [39] do not include feed-down from excited states. It is known that, among all the feed-down contributions to prompt *J*/*ψ* production from higher charmonium states, the contribution from *χ*_*c*_ mesons can be quite important (up to 30 %) and that *ψ*(2*S*) mesons also can give a sizable contribution [40–43], depending on the yields and their polarizations. The NLO NRQCD calculations [40–42] include the feed-down from *χ*_*c*_ and *ψ*(2*S*) mesons.

## Update of the *J*/*ψ* cross-section measurement

The *J*/*ψ* cross-section in *pp* collisions at $\sqrt{s} = 7~\mathrm{TeV}$ was previously measured by LHCb in 14 bins of *p*_T_ and five bins of *y* of the *J*/*ψ* meson [2]. The uncertainty on the prompt cross-section measurement is dominated by the unknown *J*/*ψ* polarization, resulting in uncertainties of up to 20 %:  where the first uncertainty is statistical, the second is systematic and the third one is due to the unknown polarization.

The previous measurement of the prompt *J*/*ψ* cross-section can be updated in the range of the polarization analysis, 2<*p*_T_<14 GeV/*c* and 2.0<*y*<4.5, by applying the measured polarization and its uncertainty to the efficiency calculation in the cross-section measurement. To re-evaluate the *J*/*ψ* production cross-section, the same data sample, trigger and selection requirements as in Ref. [2] are used. Technically the polarization correction is done by reweighting the muon angular distribution of a simulated sample of unpolarized *J*/*ψ*→*μ* ^+^ *μ* ^−^ events to reproduce the expected distribution, according to Eq. (1), for polarized *J*/*ψ* mesons. The polarization parameters *λ*_*θ*_, *λ*_*θϕ*_ and *λ*_*ϕ*_ are set to the measured values, quoted in Table 2 for each bin of *p*_T_ and *y* of the *J*/*ψ* meson.

**Fig. 7:**
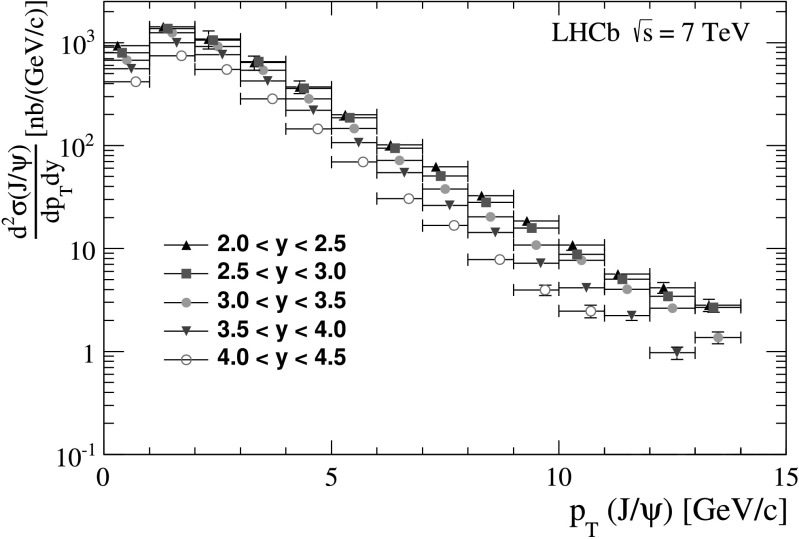
Differential cross-section of prompt *J*/*ψ* production as a function of *p*_T_ and in bins of *y*. The *vertical error bars* show the quadratic sum of the statistical and systematic uncertainties

In addition to the polarization update, the uncertainties on the luminosity determination and on the track reconstruction efficiency are updated to take into account the improvements described in Refs. [44, 45]. For the tracking efficiency it is possible to reduce the systematic uncertainty to 3 %, compared to an 8 % uncertainty assigned in the original measurement [2]. Taking advantage of the improvements described in [44] the uncertainty due to the luminosity measurement has been reduced from the 10 %, quoted in [2] to the 3.5 %. The results obtained for the double-differential cross-section are shown in Fig. 7 and reported in Table 4. The integrated cross-section in the kinematic range of the polarization analysis, 2<*p*_T_<14 GeV/*c* and 2.0<*y*<4.5, is  and for the range *p*_T_<14 GeV/*c* and 2.0<*y*<4.5, it is  For the two given cross-section measurements, the first uncertainty is statistical, the second is systematic, while the third arises from the remaining uncertainty due to the polarization measurement and is evaluated using simulated event samples. For the *p*_T_ range *p*_T_<2 GeV/*c*, where no polarization measurement exists, we assume zero polarization and assign as systematic uncertainty the difference between the zero polarization hypothesis and fully transverse (upper values) or fully longitudinal (lower values) polarization. For *p*_T_>2 GeV/*c* the uncertainties on the polarization measurement coming from the various sources are propagated to the cross-section measurement fluctuating the values of the polarization parameters in Eq. (1) with a Gaussian width equal to one standard deviation. The relative uncertainty due to the polarization effect on the integrated cross-section in 2<*p*_T_<14 GeV/*c* and 2.0<*y*<4.5 is 2.4 %. The relative uncertainty on the integrated cross-section in the range of Ref. [2], *p*_T_<14 GeV/*c* and 2.0<*y*<4.5, is reduced to 12 % (lower polarization uncertainty) and to 9 % (upper polarization uncertainty) with respect to the value published in Ref. [2].

## Conclusion

A measurement of the prompt *J*/*ψ* polarization obtained with *pp* collisions at $\sqrt{s}= 7~\mathrm{TeV}$, performed using a dataset corresponding to an integrated luminosity of 0.37 fb^−1^, is presented. The data have been collected by the LHCb experiment in the early 2011. The polarization parameters (*λ*_*θ*_,*λ*_*θϕ*_,*λ*_*ϕ*_) are determined by studying the angular distribution of the two muons from the decay *J*/*ψ*→*μ* ^+^ *μ* ^−^ with respect to the polar and azimuthal angle defined in the helicity frame. The measurement is performed in five bins of *J*/*ψ* rapidity *y* and six bins of *J*/*ψ* transverse momentum *p*_T_ in the kinematic range 2<*p*_T_<15 GeV/*c* and 2.0<*y*<4.5.

The results for *λ*_*θ*_ indicate a small longitudinal polarization while the results for *λ*_*θϕ*_ and *λ*_*ϕ*_ are consistent with zero. Although a direct comparison is not possible due to the different collision energies and analysis ranges, the measurements performed by CDF [22], PHENIX [23], HERA-B [24] and CMS [26] show no significant transverse or longitudinal polarization. Good agreement has also been found with ALICE measurements [25], performed in a *p*_T_ and rapidity range very similar to that explored by LHCb.

Our results, that are obtained for prompt *J*/*ψ* production, including the feed-down from higher excited states, contradict the CSM predictions for direct *J*/*ψ* production, both in the size of the polarization parameters and the *p*_T_ dependence. Concerning the NRQCD models, predictions from Refs. [41, 42] give the best agreement with the LHCb measurement.

This evaluation of the polarization is used to update the measurement of the integrated *J*/*ψ* production cross-section [2] in the range *p*_T_<14 GeV/*c* and 2.0<*y*<4.5, resulting in a reduction of the corresponding systematic uncertainty to 9 % and 12 %. The result is  The integrated cross-section has also been measured in the polarization analysis range 2<*p*_T_<14 GeV/*c* and 2.0<*y*<4.5:  with an uncertainty due to polarization of 2.4 %.

## Appendix

**Table 2:** Measured *J*/*ψ* polarization parameters in bins of *p*_T_ and *y* in the helicity frame. The first uncertainty is statistical (from the fit and the background subtraction) while the second is the systematic uncertainty

*p*_T_ (GeV/*c*)	*λ*	2.0<*y*<2.5	2.5<*y*<3.0	3.0<*y*<3.5	3.5<*y*<4.0	4.0<*y*<4.5
2–3	*λ*_*θ*_	−0.306±0.095±0.288	−0.207±0.010±0.101	−0.169±0.006±0.066	−0.161±0.005±0.059	−0.081±0.008±0.092
	*λ*_*θϕ*_	0.057±0.052±0.114	−0.055±0.004±0.039	−0.054±0.003±0.034	0.004±0.003±0.043	0.052±0.006±0.050
	*λ*_*ϕ*_	0.034±0.016±0.075	0.023±0.003±0.043	0.009±0.002±0.027	0.036±0.003±0.026	0.048±0.005±0.041
3–4	*λ*_*θ*_	−0.419±0.073±0.218	−0.077±0.010±0.100	−0.173±0.006±0.056	−0.149±0.006±0.054	−0.125±0.010±0.086
	*λ*_*θϕ*_	−0.055±0.044±0.094	−0.024±0.004±0.030	−0.029±0.003±0.023	0.022±0.003±0.026	0.045±0.005±0.046
	*λ*_*ϕ*_	0.021±0.016±0.045	−0.014±0.003±0.018	−0.002±0.003±0.019	0.029±0.003±0.025	0.013±0.006±0.034
4–5	*λ*_*θ*_	−0.390±0.056±0.174	−0.022±0.010±0.077	−0.149±0.007±0.050	−0.129±0.007±0.055	−0.158±0.012±0.099
	*λ*_*θϕ*_	−0.059±0.037±0.075	−0.013±0.004±0.029	−0.037±0.004±0.023	0.003±0.004±0.026	0.078±0.007±0.048
	*λ*_*ϕ*_	0.032±0.015±0.038	−0.004±0.003±0.015	−0.009±0.003±0.017	0.025±0.004±0.022	−0.015±0.008±0.031
5–7	*λ*_*θ*_	−0.126±0.037±0.133	−0.072±0.009±0.067	−0.158±0.007±0.048	−0.104±0.008±0.055	−0.045±0.013±0.098
	*λ*_*θϕ*_	−0.051±0.024±0.064	−0.010±0.004±0.026	0.007±0.004±0.022	−0.022±0.005±0.026	0.005±0.008±0.053
	*λ*_*ϕ*_	−0.016±0.010±0.031	−0.014±0.003±0.012	−0.035±0.003±0.014	0.027±0.003±0.018	0.030±0.007±0.026
7–10	*λ*_*θ*_	0.009±0.037±0.120	−0.217±0.012±0.064	−0.162±0.011±0.055	−0.042±0.013±0.066	−0.057±0.020±0.100
	*λ*_*θϕ*_	0.027±0.023±0.048	−0.016±0.005±0.026	0.029±0.005±0.022	0.006±0.007±0.028	−0.005±0.012±0.053
	*λ*_*ϕ*_	0.003±0.010±0.024	−0.008±0.004±0.011	−0.025±0.004±0.013	0.007±0.005±0.016	0.034±0.010±0.027
10–15	*λ*_*θ*_	−0.248±0.047±0.115	−0.267±0.020±0.075	−0.040±0.022±0.077	−0.076±0.028±0.082	−0.089±0.046±0.115
	*λ*_*θϕ*_	−0.088±0.027±0.054	−0.012±0.009±0.028	0.018±0.010±0.023	0.010±0.014±0.035	−0.043±0.025±0.042
	*λ*_*ϕ*_	0.009±0.014±0.029	0.008±0.007±0.013	−0.018±0.009±0.017	−0.014±0.012±0.019	−0.027±0.021±0.040

**Table 3:** Measured *J*/*ψ* polarization parameters in bins of *p*_T_ and *y* in Collins–Soper frame. The first uncertainty is statistical (from the fit and the background subtraction) while the second is the systematic uncertainty

*p*_T_ (GeV/*c*)	*λ*	2.0<*y*<2.5	2.5<*y*<3.0	3.0<*y*<3.5	3.5<*y*<4.0	4.0<*y*<4.5
2–3	*λ*_*θ*_	−0.305±0.118±0.338	−0.176±0.009±0.108	−0.130±0.004±0.058	−0.051±0.005±0.067	−0.043±0.011±0.085
	*λ*_*θϕ*_	0.152±0.044±0.158	0.114±0.006±0.058	0.102±0.004±0.035	0.098±0.003±0.036	0.037±0.005±0.050
	*λ*_*ϕ*_	−0.031±0.011±0.125	0.014±0.003±0.059	0.008±0.002±0.038	−0.001±0.002±0.031	−0.005±0.003±0.036
3–4	*λ*_*θ*_	−0.180±0.086±0.215	−0.076±0.007±0.067	−0.064±0.004±0.034	0.017±0.005±0.042	−0.001±0.011±0.070
	*λ*_*θϕ*_	0.223±0.042±0.095	0.090±0.006±0.047	0.109±0.004±0.031	0.081±0.004±0.032	0.015±0.006±0.049
	*λ*_*ϕ*_	−0.070±0.014±0.065	−0.027±0.004±0.036	−0.033±0.003±0.028	−0.017±0.004±0.026	−0.049±0.005±0.040
4–5	*λ*_*θ*_	−0.084±0.068±0.171	−0.000±0.007±0.040	−0.035±0.005±0.030	0.031±0.006±0.037	0.051±0.012±0.071
	*λ*_*θϕ*_	0.240±0.041±0.092	0.067±0.006±0.041	0.081±0.004±0.027	0.065±0.004±0.030	−0.028±0.008±0.052
	*λ*_*ϕ*_	−0.104±0.017±0.055	−0.042±0.005±0.032	−0.050±0.005±0.027	−0.033±0.005±0.029	−0.095±0.007±0.047
5–7	*λ*_*θ*_	−0.110±0.037±0.081	0.008±0.006±0.032	0.005±0.005±0.027	0.054±0.006±0.033	0.089±0.012±0.072
	*λ*_*θϕ*_	0.160±0.029±0.070	0.056±0.005±0.032	0.041±0.004±0.023	0.063±0.004±0.028	−0.000±0.008±0.053
	*λ*_*ϕ*_	−0.068±0.014±0.051	−0.056±0.005±0.031	−0.085±0.005±0.026	−0.051±0.005±0.031	−0.056±0.008±0.052
7–10	*λ*_*θ*_	0.079±0.032±0.061	0.035±0.009±0.035	0.032±0.009±0.030	0.031±0.011±0.036	0.072±0.020±0.071
	*λ*_*θϕ*_	0.014±0.028±0.061	0.073±0.006±0.026	0.036±0.005±0.023	0.022±0.007±0.029	0.007±0.013±0.045
	*λ*_*ϕ*_	−0.074±0.018±0.053	−0.078±0.007±0.032	−0.076±0.007±0.029	−0.027±0.009±0.036	−0.022±0.014±0.055
10–15	*λ*_*θ*_	0.064±0.037±0.076	0.099±0.016±0.046	−0.004±0.018±0.044	−0.009±0.024±0.050	0.019±0.042±0.086
	*λ*_*θϕ*_	0.105±0.033±0.057	0.070±0.010±0.024	0.004±0.010±0.024	0.021±0.014±0.028	0.033±0.026±0.041
	*λ*_*ϕ*_	−0.093±0.026±0.059	−0.108±0.013±0.040	−0.024±0.013±0.040	−0.024±0.017±0.048	−0.084±0.030±0.064

**Table 4:** Double-differential cross-section *d* ^2^ *σ*/*dp*_T_ *dy* in nb/(GeV/*c*) for prompt *J*/*ψ* production in bins of *p*_T_ and *y*, with statistical, systematic and polarization uncertainties

*p*_T_ (GeV/*c*)	2.0<*y*<2.5	2.5<*y*<3.0	3.0<*y*<3.5	3.5<*y*<4.0	4.0<*y*<4.5
2–3	1083±18±64±210	1055±8±61±47	918±6±53±28	762±5±46±23	549±5±36±27
3–4	639±9±41±93	653±5±39±28	541±4±32±17	422.9±3.4±26.2±12.9	284±3±19±16
4–5	370±5±24±46	359.1±3.1±22.3±14.1	285.1±2.4±17.7±8.5	219.1±2.3±13.9±6.7	145.4±2.4±9.2±8.7
5–6	199.0±3.0±13.8±17.4	185.9±2.0±12.2±6.2	146.4±1.7±9.3±4.2	107.2±1.4±7.5±3.2	69.2±1.5±4.4±3.5
6–7	101.2±1.9±7.3±8.0	94.1±1.3±6.4±2.9	71.7±1.1±4.8±1.9	54.6±1.0±3.5±1.6	30.6±1.0±1.9±1.4
7–8	62.2±1.4±4.1±4.6	50.6±0.9±3.7±1.7	37.8±0.7±2.4±1.2	26.2±0.6±1.7±0.9	16.71±0.69±1.06±0.92
8–9	32.5±0.9±2.1±2.2	28.1±0.7±1.8±0.9	20.3±0.5±1.3±0.6	14.3±0.5±0.9±0.5	7.78±0.43±0.49±0.39
9–10	18.5±0.7±1.2±1.3	15.8±0.5±1.0±0.5	10.8±0.4±0.7±0.3	7.18±0.32±0.46±0.22	3.96±0.31±0.25±0.24
10–11	10.8±0.5±0.7±0.9	8.7±0.4±0.6±0.3	7.70±0.34±0.50±0.31	4.15±0.24±0.27±0.18	2.47±0.25±0.16±0.18
11–12	5.65±0.32±0.37±0.41	5.04±0.26±0.32±0.18	4.03±0.23±0.26±0.13	2.24±0.17±0.14±0.08	–
12–13	4.16±0.27±0.27±0.32	3.42±0.23±0.22±0.14	2.64±0.18±0.17±0.09	0.97±0.11±0.06±0.04	–
13–14	2.82±0.26±0.19±0.21	2.68±0.20±0.17±0.11	1.37±0.15±0.09±0.06	–	–

## Footnotes

1 In the ALICE measurement the *J*/*ψ* from *b* decays are also included.

2 The signal window and the sum of the sideband regions have the same width.
